# Exosomes and Obesity-Related Insulin Resistance

**DOI:** 10.3389/fcell.2021.651996

**Published:** 2021-03-18

**Authors:** Li-Min Lei, Xiao Lin, Feng Xu, Su-Kang Shan, Bei Guo, Fu-Xing-Zi Li, Ming-Hui Zheng, Yi Wang, Qiu-Shuang Xu, Ling-Qing Yuan

**Affiliations:** ^1^National Clinical Research Center for Metabolic Disease, Hunan Provincial Key Laboratory of Metabolic Bone Diseases, Department of Endocrinology and Metabolism, The Second Xiangya Hospital, Central South University, Changsha, China; ^2^Department of Radiology, The Second Xiangya Hospital, Central South University, Changsha, China

**Keywords:** exosomes, insulin resistance, obesity, inflammation, mesenchymal stem cell

## Abstract

Exosomes are extracellular vesicles, delivering signal molecules from donor cells to recipient cells. The cargo of exosomes, including proteins, DNA and RNA, can target the recipient tissues and organs, which have an important role in disease development. Insulin resistance is a kind of pathological state, which is important in the pathogeneses of type 2 diabetes mellitus (T2DM), gestational diabetes mellitus and Alzheimer’s disease. Furthermore, obesity is a kind of inducement of insulin resistance. In this review, we summarized recent research advances on exosomes and insulin resistance, especially focusing on obesity-related insulin resistance. These studies suggest that exosomes have great importance in the development of insulin resistance in obesity and have great potential for use in the diagnosis and therapy of insulin resistance.

## Exosome

**FIGURE 1:**
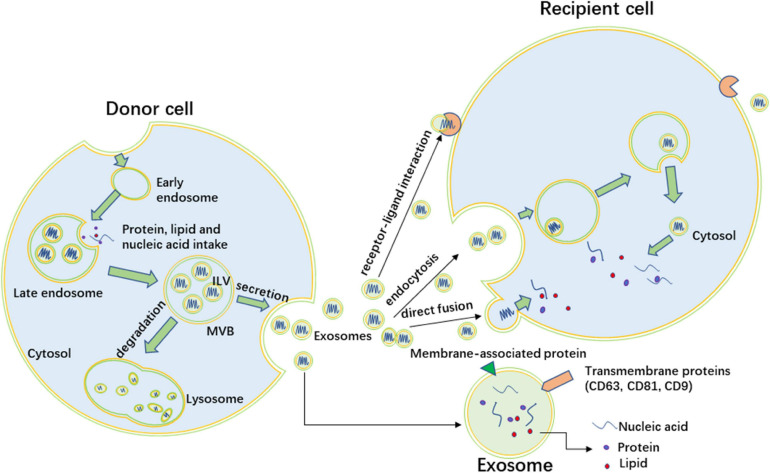
The formation of exosomes and intercellular interaction. Exosomes originate as intraluminal vesicles (ILVs) that form by inward budding of the limiting membrane of early endosomes. The endosomes mature into multivesicular bodies (MVBs) which fuse with the plasma membrane to release exosomes. Other MVBs fuse with lysosome, and the ILVs are degraded by lysosomes. Exosomes contain nucleic acid, protein, and lipid, the membrane of exosomes also include membrane proteins of endosomes. CD63, CD81, and CD9 are common surface biomarkers of exosomes. Exosomes target recipient cells through three ways, including direct fusion, endocytosis, and receptor-ligand interaction.

Exosomes, which are considered extracellular vesicles (EVs), are cup-shaped structures with a diameter of 30–150 nm and a lipid bilayer membrane (Wortzel et al., 2019). The information molecules carried by exosomes include lipids (Carayon et al., 2011), proteins (Li et al., 2017) and nucleic acids (Kamalden et al., 2017), and these types of cargo are protected from degradation by the lipid bilayer membrane and can be transported from donor cells to recipient cells (Figure 1). There are many marker molecules to determine exosomes, including CD81, CD9, CD63, heat-shock proteins (HSP60, HSP70, and HSP90), ALG-2 (apoptosis-linked gene 2)-interacting protein X (ALIX), and tumor susceptibility gene 101 (TSG101) (Lou et al., 2017), and glucose-regulated protein 94 (Grp94) as a negative marker (Gemel et al., 2019). As the new carrier of information exchange between cells, exosomes can be secreted by different types of cells, such as hepatocytes (Fang et al., 2018), adipocytes (Yu et al., 2018), skeletal muscle cells (Nie et al., 2019), vascular smooth muscle cells (Kapustin et al., 2015) and stem cells (Willis et al., 2018). Exosomes have been detected in various bodily fluids, including plasma, saliva, breast milk, sweat, tears, and urine (Lasser et al., 2011; McKiernan et al., 2018; Wu and Liu, 2018; Inubushi et al., 2020). What’s the precise function of exosomes? Initially, exosomes were described as “garbage dumpsters,” which took the useless or harmful intracellular substances out of cells. Recently, exosomes were defined as “signal boxes,” which delivered messages between the cells or organs (Xie et al., 2019). Now, more and more studies have elucidated the functions of exosomes. Exosomes can participate in both pathological and physiological processes, including angiogenesis (Zeng et al., 2018), vascular calcification (Xu F. et al., 2020), immune inflammation (Pan et al., 2019), apoptosis (Guay et al., 2019), fibrosis (Seo et al., 2016), tumor development (Xue et al., 2017), senescence (Zhang et al., 2017; Xu F. et al., 2020), tissue repair (Dinh et al., 2020) and insulin resistance (Yu et al., 2018). Our present review will focus on the exosome’s effect on insulin resistance.

## Insulin Physiology and Insulin Resistance

As a key regulatory factor of glucose metabolism, insulin is secreted by pancreatic β cells following elevations in blood glucose (Halperin et al., 2012). Indeed, the actions of insulin mainly contribute to glucose uptake by skeletal muscle and adipose tissue and reduce liver gluconeogenesis and glycogenolysis (Titchenell et al., 2017; Tokarz et al., 2018). Insulin also participates in protein metabolism in skeletal metabolism and lipid storage in adipose tissue (Fu et al., 2015). Recently, scientists have found that the brain was also a target tissue of insulin (Honkala et al., 2018). Insulin signaling has many complex branches, and each branch can present a kind of physiologic function. The phosphorylation of Akt takes part in most of the insulin signaling pathways, so we usually regard the level of p-AKT as a marker of insulin sensibility (Friedrichsen et al., 2010).

Insulin resistance is one of the important pathogenetic mechanisms of type 2 diabetes mellitus (T2DM) (Stenvers et al., 2018). Insulin resistance also takes a part in the development of gestational diabetes mellitus (GDM) and Alzheimer’s disease (Benhalima et al., 2019; Wakabayashi et al., 2019). Insulin resistance refers to an impairment in the biological effects of insulin on target tissues, such as the ability to promote glucose uptake and inhibit the breakdown of glycogen in adipocytes and skeletal muscle cells. In addition to glucose metabolism, insulin is also involved in protein and lipid metabolism. In fact, we usually define the impairment of insulin effect on glucose metabolism as insulin resistance. When insulin resistance occurs in the body, the compensatory secretion of insulin will cause hyperinsulinaemia, resulting in a series of pathophysiological changes, such as pancreatic islet function impairment, increasing nutrient consumption and hyperlipidemia, cardiovascular damage, and finally leading to a variety of metabolic diseases, such as diabetes mellitus, obesity (Templeman et al., 2017) and diabetic cardiomyopathy (Jia et al., 2016). There are several methods to determine insulin resistance, such as the hyperinsulinaemic euglycaemic clamp, oral glucose tolerance test (OGTT) and Homeostatic Model Assessment for Insulin Resistance (HOMA-IR).

Among all the reasons leading to insulin resistance, obesity is one of the most significant causes of insulin resistance (Day et al., 2017). The sign of obesity is the expansion of adipose tissue, which is mainly reflected in the increase of subcutaneous adipose tissue. Subcutaneous adipose tissue is the largest warehouse of adipose tissue in humans and the preferred place for storing excess fat. However, the expanding ability of subcutaneous adipose tissue is limited. When its storage capacity is exceeded, lipids are stored in other metabolically more harmful ectopic tissues, such as the liver and skeletal muscle (Gustafson et al., 2015). Adipose tissues can store triglycerides and set free fatty acids and glycerol (Mundi et al., 2014). Free fatty acids can contribute to insulin insensitivity (Vlavcheski and Tsiani, 2018). White adipose tissue plays the most important role in storing fatty acids. By enhancing lipid synthesis or limiting lipolysis, white adipocytes store lipids to prevent toxic lipid accumulation in the liver and skeletal muscle (Czech, 2020). One study showed that the percentage of fat stored in the white adipose tissue of obese people decreased significantly, because the storage of lipids in non-adipose tissues were promoted (McQuaid et al., 2011). In contrast, brown/beige adipocytes were active in the direct uptake of glucose in response to β-adrenergic signaling and insulin, increasing energy consumption (Czech, 2020). Obesity has a detrimental effect on the function of each type of adipocyte, leading to insulin resistance (Czech, 2020). In addition to its function of storing fat, adipose tissue is also a very important endocrine organ. Adipokines, a general term for hormones secreted by adipocytes, are divided into two groups, which promote insulin actions or inhibit insulin actions, respectively (Guo et al., 2017). Leptin and adiponectin are adipokines that increase insulin sensitivity (Ayina et al., 2016). Adipose tissues can release tumor necrosis factor-α (TNF-α) and interleukin-6 (IL-6) as inhibitory adipokines, which inhibit insulin action. In the process of adipose tissue expansion in obese people, adipose tissue produces chronic inflammation, and many M1 macrophages are recruited into adipose tissue and produce inflammatory factors. These inflammatory factors lead to insulin resistance in adipose tissues and act on other tissues to induce insulin resistance (Olefsky and Glass, 2010). All in all, adipose organ dysfunction is a key cause in the development of insulin resistance.

## Exosomes in Obesity-Related Insulin Resistance

Obesity is the most significant risk factor for insulin resistance, but the specific pathogenesis of insulin resistance needs further study. Recently, exosomes have been proven to act as communicators between cells, which have attracted the attention of many scientists. Exosomes contain many kinds of cargo, which can influence the function of recipient cells. Researchers have revealed that exosomes participate in many kinds of disease development processes, such as liver and kidney diseases (Babuta et al., 2019; Liu et al., 2020). At the same time, the role of exosomes in insulin resistance has been studied, with many studies focusing on the effect of exosomes in obesity-related insulin resistance.

### The Role of Exosomes From Adipose Tissues in Obesity-Related Insulin Resistance

**FIGURE 2:**
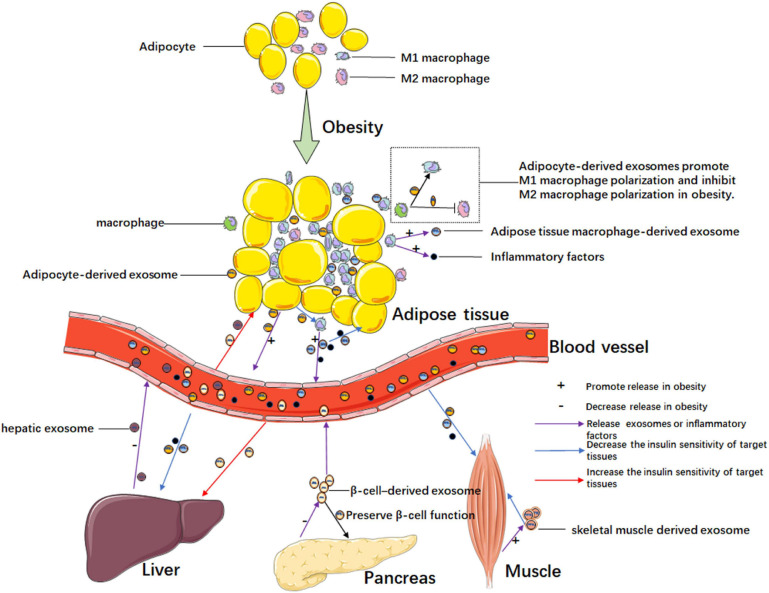
Exosome-mediated intercellular communication in obesity-related insulin resistance. Chronic inflammation exits in adipose tissue in obesity. Adipocyte-derived exosomes promote the polarization of M1 macrophages which secretes pro-inflammatory cytokines and exosomes, and the adipose tissue macrophage-derived exosomes can promote insulin resistance in adipocytes. In obesity, exosomes from adipose tissue, liver, pancreas, and muscle, mediating intra-organ cross talks or inter-organ cross talks by blood circulation. In obesity, these organs or tissues increase the secretion of exosomes that promote insulin resistance or decrease the secretion of exosomes that ameliorate insulin resistance.

Since adipose tissue plays a key role in insulin resistance, exosomes secreted from adipose tissue may be a kind of media in this process. The chronic inflammation of adipose tissue is an especially significant inducing factor in the development of insulin resistance, and the chronic inflammation manifests as macrophage infiltration. In recent years, interest has been emerging in the research of adipocyte-derived exosomes of chronic inflammation in adipose tissue (Figure 2). Deng et al. found exosomes secreted by the adipose tissue of ob/ob mice (obese model) induced the activation of macrophages through the TLR4/TRIF pathway, and the retinol-binding protein-4 (RBP4) in these exosomes played a role in the induction of macrophage activation. Furthermore, the obesity-related exosomes homed the macrophage to the liver and adipose tissues, in which macrophages secreted TNF-α and IL-6 to result in insulin resistance. They also confirmed the exosome-mediated macrophage impaired insulin action of myocytes. This is the first research discovering the role of exosomes in obesity-related macrophage-mediated insulin resistance (Deng et al., 2009). Song and co-workers confirmed that Sonic hedgehog (Shh) from insulin resistance adipocyte-derived exosomes (IRADEs) was the key regulator mediating M1 macrophage polarization through Ptch/PI3K signaling. They uncovered the exosomes from the IRADE-treated macrophages could contribute to insulin resistance in adipose tissues by decreasing the expression of insulin receptor substrate-1 (IRS-1) and hormone-sensitive lipase (HSL) expression. The authors gave us a new target, Shh, to inhibit the development of insulin resistance (Song et al., 2018). Adipose tissue macrophage (ATM)-derived exosomes from obese mice induced insulin resistance. However, the exosomes from lean ATMs mitigated the insulin resistance in obese mice without changing the weight of the mice. The authors revealed the key point of the effect of ATM-derived exosomes in modulating insulin sensitivity was miR-155 by targeting peroxisome proliferator-activated receptor γ (PPARγ). Furthermore, miR-155 knockout mice were much more insulin sensitive than their obese wildtype mice control counterparts (Ying et al., 2017). Similarly, Liu et al. (2019) found that exosomal miR-29a from ATMs was highly expressed in obese mice and could be transferred to adipocytes, myocytes and hepatocytes to induce insulin insistence by targeting PPARδ. A study showed exosomes from lipopolysaccharide (LPS)-activated macrophages could change the adipocyte gene expression associated with inflammation. However, the exosomes could not influence insulin-dependent glucose uptake (De Silva et al., 2018). Likewise, Pan and colleagues also confirmed that adipose tissue-related macrophage polarization was of great importance in insulin resistance. Exosomal miR-34a secreted by adipocytes inhibited M2 macrophage polarization by targeting Krüppel-like factor 4 (Klf4), which promoted M2 macrophage polarization and monocyte differentiation. The authors also verified that the altered miR-34a/Klf4 axis in visceral fat was closely associated with insulin resistance in obese subjects (Pan et al., 2019). In short, exosomes from adipocytes can increase the M1 macrophage, which secretes inflammatory cytokines, leading to insulin resistance. Apart from the inflammatory factors, ATMs also secrete exosomes to regulate insulin sensitivity. Furthermore, exosomes from macrophages can modulate adipocyte metabolism. Adipocyte-derived exosomes also decrease M2 macrophage levels, which is a kind of cell that inhibits inflammation.

Apart from participating in inflammation-induced insulin resistance in adipose tissues, exosomes from adipocytes also have crosstalk with other organs, inducing obesity-related insulin resistance (Figure 2). A previous study revealed crosstalk between adipose tissue and skeletal muscle tissue in obesity-related insulin resistance, and authors found that adipocyte-derived exosomal miR-27a decreased the expressions of IRS-1 and glucose transporter GLUT4 in skeletal muscle tissue by targeting PPARγ. The authors confirmed exosomal miR-27a was derived from adipocytes, not macrophages and skeletal muscle cells (Yu et al., 2018). Dang et al. revealed that exosomes derived from adipocytes in ob/ob mice and B6 (C57BL/6j) mice fed with a high-fat diet (HFD) could decrease insulin sensitivity by affecting phosphorylation levels of AKT and glucose uptake of AML12 cells. Furthermore, the inhibition of insulin function was mainly caused by obesity in the adipose tissue of ob/ob mice, which secreted miR-141-3p deficiency exosomes taken up by hepatocytes. The author confirmed that miR-141-3p could target phosphatase and tensin homolog deleted on chromosome 10 (PTEN), a negative regulator of the PI3K/AKT signaling pathway (Dang et al., 2019). In a previous study, human adipose tissue-derived EVs were revealed to inhibit hepatic insulin signaling by reducing the p-AKT levels. However, the adipose tissue-derived EVs did not have a significant effect on skeletal muscle cells compared to hepatocytes. Authors found that the level of monocyte chemoattractant protein-1 in subcutaneous adipose tissue-derived EVs and levels of IL-6 and macrophage migration inhibitory factor of omental adipose tissue-derived EVs were associated with AKT phosphorylation in hepatocytes (Kranendonk et al., 2014). Li et al. showed adipose-specific knockdown of Sirtuin 1 (Sirt1) contributed to obesity and insulin resistance by stimulating exosomes secretion in an autophagy-dependent manner, and adipose Sirt1 deficiency-induced exosomes affected insulin sensitivity via the TLR4/NF-κB signaling pathway in adipose tissues (Li F. et al., 2019). As adipose tissue expansion could lead to local tissue hypoxia and inflammation in obesity, Mleczko et al. revealed that the exosomes from hypoxic adipocytes impaired insulin-stimulated glucose uptake by reducing the phosphorylation of AKT. The authors also confirmed exosomes from plasma obtained from obese women could reduce insulin-stimulated glucose transport. Interestingly, the inhibiting effect of the exosomes could be restored by heating the hypoxic exosomes preparation to 40°C for 30 min prior to the treatment of cells. Since the effect of hypoxic EVs on glucose transport was thermolabile, the authors guessed enzymatic activity might be responsible for this effect. Then, they found PTEN, a protein phosphatase that reduced phosphorylation of AKT existed in EVs from hypoxic 3T3L1 adipocytes. However, they did not find the precise molecular mechanisms (Mleczko et al., 2018). Hubal et al. (2017) found that gastric bypass bariatric surgery could not only reduce weight and insulin resistance but also change the microRNA content of circulating adipocyte-derived exosomes isolated from the peripheral blood. Among the altered microRNAs of exosomes, 29 microRNAs associated with insulin resistance changed after surgery; likewise, 48 microRNAs targeting 78 mRNAs were significantly correlated to branched-chain amino acid levels, which are linked to insulin dysregulation (Hubal et al., 2017). These studies suggested that exosomes from adipocytes could have a function in distant organs and take part in obesity-related insulin resistance.

### The Role of Exosomes Derived From Tissues Other Than Adipose Tissue in Obesity-Related Insulin Resistance

In obesity, other organs could also produce exosomes that are associated with insulin resistance (Figure 2). A study by Wu et al. (2020) revealed that hepatic exosome-derived miR-130a-3p participated in lipid and glucose metabolism by targeting adipocytes. In HFD-induced mice, miR-130a-3p knockout mice had the highest blood lipid index and a higher blood glucose level compared to those in miR-130a-3p overexpressed and wild-type mice. Authors found hepatic exosome-derived miR-130a-3p suppressed adipogenesis mainly by downregulating the expression of fatty acid synthetase (FASN) and PPARγ at the protein level. Besides, the authors revealed that miR-130a-3p could target PH domain leucine-rich repeat protein phosphatase 2 (PHLPP2), increase the levels of p-AKT and p-AS160, and finally promote GLUT4 transportation (Wu et al., 2020). In a previous study, the authors confirmed the effect of skeletal muscle-derived exosomes during lipid-induced insulin resistance. In addition to insulin resistance, lipid treatment led skeletal muscle to produce more exosomes, which could increase the AKT content of recipient muscle cells, and regulate the expression of genes having to do with the cell cycle and muscle differentiation in recipient myotubes. The exosomes from lipid-treated skeletal muscle also induced myoblast proliferation. Authors also revealed that exosomes transferred lipids between muscle cells (Aswad et al., 2014). As the insulin-secreting organ, the islets are also involved in regulating insulin sensitivity. Xu et al. revealed that pancreatic β cell miR-26a improved insulin sensitivity and preserved β cell function. After confirming exosomal miR-26a was reduced in obese humans and mice, especially in the islets, the authors generated conditional transgenic mice expressing miR-26a under the control of the rat insulin promoter to increase expression of miR-26a in β cells. The results showed that the exosomal miR-26a secreted from β cells could increase insulin sensitivity in peripheral tissues by regulating metabolism-related gene expression, and the miR-26a could also decrease glucose-stimulated insulin secretion (GSIS) by impairing actin cytoskeleton remodeling. In addition, miR-26a ameliorated compensatory β cell hyperplasia by decreasing β cell replication induced by excess nutrition (Xu H. et al., 2020). In an interesting study, Wang et al. found pancreatic cancer-derived exosomes also inhibited glucose intake in C2C12 myotube cells through the PI3K/AKT/FoxO1 pathway. The increase of FoxO1 caused by pancreatic cancer-derived exosomes contributed to inhibiting the translocation of GLUT4 to the plasma membrane. Although the authors showed exosomal miRNA might be involved in this process through microRNA microarray analysis, they did not confirm the concrete molecular mechanism (Wang et al., 2017). A study by Choi et al. (2015) confirmed that EVs from gut microbes induced by a high fat diet (HFD) could impair insulin signaling and glucose metabolism both *in vitro* and *in vivo*. They observed that the HFD changed the bacteria in the gut. However, the gut bacteria did not infiltrate through the gut to other organs, but the gut microbe-derived EVs caused insulin resistance by infiltrating the gut barrier and targeting other organs (Choi et al., 2015). In a previous study, researchers determined the circulating exosome miRNA profile in diet-induced central obesity mice and found an increase in miR-122, miR-192, miR-27a-3p, and miR-27b-3p. As the exosomes from obese mice could induce insulin resistance, authors used exosomes transfected with obesity-associated miRNA mimic injected into lean mice to exclude other sources of variation. The results showed that mimic treatment targeted PPARγ to induce inflammation and hepatic steatosis in epididymal white adipose tissue (eWAT), both of which are known to participate in glucose intolerance and dyslipidemia, and the effects of mimic treatment could be reverted by the lipolysis inhibitor acipimox or the PPARα agonist fenofibrate (Castaño et al., 2018). Likewise, a clinical study showed that the number of circulating EVs was strongly associated with obesity and lipid and glucose metabolism. Furthermore, some EVs were confirmed from adipocytes and hepatocytes, which are major metabolic cells (Kobayashi et al., 2018).

Above all, insulin resistance is a complicated pathological state, which affects various organs. Furthermore, exosomes play a pivotal role in the development of obesity-related insulin resistance. Above all, the function of exosomal contents can be seen in Table 1.

**TABLE 1:** Role of exosomes in obesity-related insulin resistance.

Source	Contents	Functions	Level	References
Adipocytes	RBP4	Activation of macrophage impairing glucose uptake and the insulin response depending on the TLR4 pathway and inducing TNF-α and IL-6	Overexpress	Deng et al., 2009
Insulin resistance adipocytes	Sonic Hedgehog (Shh)	Mediating M1 macrophage polarization through Ptch/PI3K signaling and educating macrophage which produces exosomes causing insulin resistance by decreasing the expression of IRS-1 and HSL expression	Overexpress	Song et al., 2018
Adipocytes	microRNA-34a	Inhibiting M2 macrophage polarization by targeting Klf4	Overexpress	Pan et al., 2019
Adipose tissue macrophages	miR-155	Impairing insulin sensitivity by targeting PPARγ	Overexpress	Ying et al., 2017
Adipose tissue macrophages	miR-29a	Transferring to adipocytes, myocytes, and hepatocytes to induce insulin insistence by targeting PPAR-δ	Overexpress	Liu et al., 2019
Adipocytes	miR-27a	Decreasing the expressions of IRS-1 and GLUT4 in skeletal muscle tissue by targeting PPARγ	Overexpress	Yu et al., 2018
Adipocytes	miR-141-3p	Increasing PI3K/AKT signaling pathway by targeting PTEN in hepatocytes	Downexpress	Dang et al., 2019
Adipocytes	Sirt1	Decreasing insulin resistance by reducing TLR4/NF-κB signaling pathway	Downexpress	Li F. et al., 2019
Hypoxic adipocytes	–	Impairing insulin-stimulated glucose uptake by reducing AKT phosphorylation	–	Mleczko et al., 2018
Hepatocytes	miR-130a-3p	Suppressing adipogenesis by downregulating the expression of FASN and PPARγ at the protein level and increasing the level of P-AKT and P-AS160 by targeting PHLPP2	Downexpress	Wu et al., 2020
Pancreatic β cells	microRNA-26a	Increasing insulin sensitivity in peripheral tissues, decreasing glucose-stimulated insulin secretion (GSIS) by impairing actin cytoskeleton remodeling and preserving β cell function	Downexpress	Xu H. et al., 2020
Gut microbes	–	Inducing insulin resistance by infiltrating the gut barrier and targeting other organs	–	Choi et al., 2015
Serum	miR-122, miR-192, miR-27a-3p, and miR-27b-3p	Targeting Pparg to induce eWAT Inflammation and hepatic steatosis	Overexpress	Castaño et al., 2018

## Exosomes as Biomarkers for Insulin Resistance

The OGTT and HOMA-IR are useful to test for insulin resistance, although the hyperinsulinaemic euglycaemic clamp is regarded as the gold standard for the diagnosis of insulin resistance diagnosis. However, it can’t be used as a routine test. Recently, many biomarkers for insulin resistance have been revealed. Adipokines could be regarded as biomarkers for insulin resistance, including adiponectin, RBP4, chemerin, and adipocyte fatty acid-binding protein (A-FABP) (Huang et al., 2013; Li et al., 2018; Li X. et al., 2019; Frithioff-Bojsoe et al., 2020). Fibroblast growth factor 21 and fetuin-A have been found as biomarkers for insulin resistance (Shim et al., 2017; Xu et al., 2018). Many studies have shown that myokines, such as IL-6, irisin and myostatin, serve as biomarkers for insulin resistance (Mauer et al., 2014; Steculorum et al., 2016; Mazur-Bialy, 2019).

The release of exosomes depends on the state of the human body. What’s more, exosomes can protect the cargo from degradation by the lipid bilayer membrane. Therefore, collecting exosomes is a potential promising diagnostic method for diseases. Exosomes were found in various bodily fluids, such as plasma, saliva, breast milk, sweat, tears, and urine. Therefore, exosomes can easily be obtained from bodily fluids. It has been reported that exosomes are of great significance for fluid biopsy in many kinds of diseases, such as breast cancer (Hannafon et al., 2016), hepatocellular carcinoma (Sohn et al., 2015), prostate cancer (Bhagirath et al., 2018), osteoarthritis (Zhao and Xu, 2018) and atherosclerosis (Lu et al., 2019). Likewise, using exosomes for the early diagnosis of insulin resistance is a promising method. Sharma et al. reported that the phosphoenolpyruvate carboxykinase in urine exosomes reflected gluconeogenesis of the kidney. By determining phosphoenolpyruvate carboxykinase, urine exosomes, as a non-invasive marker, contribute to discovering impairment in gluconeogenesis and early insulin resistance in humans (Sharma et al., 2020). A clinical study showed that circulating miRNAs in the EVs of human plasma could be biomarkers for insulin resistance phenotypes in obesity. Authors found that four miRNAs (let-7b, miR-144-5p, miR-34a, and miR-532-5p) were strongly predictive of insulin resistance (Jones et al., 2017). Another study by Katayama et al. (2019) revealed that miRNA expression profiles in exosomes, rather than in serum, had a significant difference between normal glucose tolerance and patients with T2DM. The increase in circulating exosomal miR-20b-5p was confirmed to impair insulin signaling in human skeletal muscle by targeting AKTIP and STAT3 (Katayama et al., 2019). Brain insulin resistance exists in Alzheimer’s disease. A study showed neurally derived blood exosomes were used to track the dysfunctional phosphorylation type 1 insulin receptor, which could happen in preclinical Alzheimer’s disease (Kapogiannis et al., 2015). Mullins et al. (2017) found that neural origin plasma exosomes labeled by L1CAM contained higher pSer312-IRS-1 (ineffective insulin signaling) and lower p-panTyr-IRS-1 (effective insulin signaling) in Alzheimer’s disease. Furthermore, they revealed that higher pSer312-IRS-1 levels were positively related to greater brain atrophy in Alzheimer’s disease and p-panTyr-IRS-1 levels had the opposite effect (Mullins et al., 2017). In a word, exosomes will be the most promising bodily fluid biopsy. As shown in Table 2, some potential exosomal biomarkers can be used to track insulin resistance.

**TABLE 2:** Potential biomarkers of exosomes in insulin resistance.

Source	Potential biomarker	Express level	References
Urine exosomes	Phosphoenolpyruvate carboxykinase	Up	Sharma et al., 2020
Plasma exosomes	Let-7b, miR-144-5p, miR-34a, and miR-532-5p	Up	Jones et al., 2017
Serum exosomes	miR-20b-5p	Up	Katayama et al., 2019
Neural origin plasma exosomes	pSer312-IRS-1 and p-panTyr-IRS-1	higher pSer312-IRS-1 and lower p-panTyr-IRS-1	Mullins et al., 2017

## Mesenchymal Stem Cells-Derived Exosomes in Insulin Resistance

Mesenchymal stem cells (MSCs) are multipotent cells that can be self-renewing, including human umbilical cord mesenchymal stem cells, bone marrow mesenchymal stem cells and adipose-derived stem cells. Recently, stem cell therapy has been a promising strategy for diseases, such as liver disease (Zhao L. et al., 2018), multiple sclerosis (Muraro et al., 2017; Atkins, 2019), leukemia (Shem-Tov et al., 2020) and diabetic retinopathy (Gaddam et al., 2019). However, whether stem cell transplantation contributes to the emergence of tumors remains unknown. It has been found that stem cells have an effect on tissue-resident recipient cells by paracrine mechanisms (Liang et al., 2014). It has been reported that exosomes secreted by mesenchymal stem cells have the properties of their parent cells, such as regulation of cell migration and proliferation (McBride et al., 2017; Chew et al., 2019), immunomodulation (Willis et al., 2018), tissue regeneration (Chew et al., 2019) and anti-inflammatory (Xia et al., 2019) effects.

**FIGURE 3:**
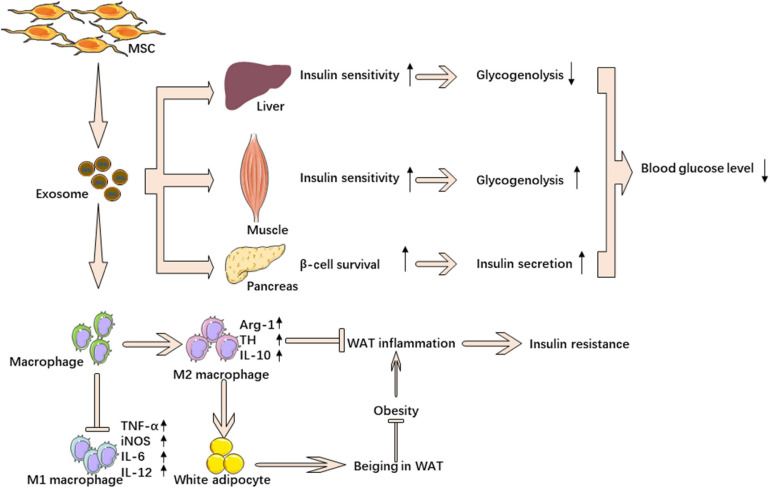
MSC-derived exosomes ameliorate insulin resistance. MSC-derived exosomes down regulate blood glucose through reverse insulin resistance in insulin target tissue and relieve β cell destruction. MSC-derived exosomes also ameliorate insulin resistance by promoting M2 macrophage polarization and inhibiting M1 macrophage polarization.

Several researchers investigated the effect of mesenchymal stem cell-derived exosomes in insulin resistance. Sun et al. showed that exosomes from human umbilical cord mesenchymal stem cells (hucMSC-ex) could have a therapeutic effect on T2DM. They found that injection of hucMSC-ex significantly ameliorated hyperglycemia in rats with T2DM. HucMSC-ex could increase insulin sensitivity by increasing the activation of p-IRS-1 and p-AKT and inhibiting the secretion of pro-inflammatory cytokines, which could inhibit the activation of the insulin signaling pathway. These exosomes could promote glucose uptake and glycolysis in skeletal muscle by affecting membrane translocation of GLUT4 and glucose metabolism-related enzymes. Activating insulin signaling, hucMSC-ex increased expression of p-GSK3β and glycogen synthase to improve glycogen synthesis in the liver. Furthermore, hucMSC-ex could not only promote the secretion of insulin but also inhibit STZ-induced β cell apoptosis (Sun et al., 2018). However, this research didn’t explain which kind of substance contained in hucMSC-ex played a role in the process. In a previous study, Su et al. (2019) found exosomes from bone marrow mesenchymal stem cells (BM-MSCs) of aged mice impaired insulin sensitivity both *in vitro* and *in vivo*. After a miRNA microarray analysis, the authors paid attention to miR-29b-3p, which was significantly higher in exosomes secreted by the BM-MSCs of aged mice compared to that released by the BM-MSCs of young mice. They revealed that Sirt1 was downstream of miR-29b-3p and regulated insulin sensitivity. Injection of the BM-MSC-specific nanocomplex/aptamer-agomiR-29b-3p to the bone marrow cavity could impair insulin sensitivity in young mice. On the contrary, BM-MSC-specific nanocomplex/aptamer-antagomiR-29b-3p mitigated aging-associated resistance in old mice. However, they did not find strong evidence showing BM-MSC-derived exosomes affect aging-associated degeneration of pancreatic function (Su et al., 2019). Zhao et al. found that exosomes from adipose-derived stem cells (ADSCs) were taken up by macrophages. Furthermore, these exosomes carried active STAT3, which could promote arginase-1 expression in macrophages to induce anti-inflammatory M2 phenotypes. ADSC-derived exosomes mitigated diet-induced obesity and improved glucose tolerance and insulin sensitivity. Besides, the authors found that ADSC-derived exosomes could inhibit adipocyte hypertrophy and promote the occurrence of brown-like fats. More interestingly, the results showed that macrophages treated by ADSC-derived exosomes could induce ADSC proliferation by secreting lactate (Zhao H. et al., 2018). Taken together, stem cell-derived exosomes could increase insulin sensitivity by promoting insulin signaling and decreasing adipose tissue-related inflammation (Figure 3).

## Summary and Perspectives

Obesity is of great importance in the development of insulin resistance. The chronic inflammation in adipose tissues is the main cause of insulin resistance in obesity, and great importance in the development of inflammation has been attached to macrophages. Exosomes, as the mediators, delivering the contents from parent cells to recipient cells, affect the pathophysiology of human beings. In this review, we summarized a lot of exosome-associated research, which mainly focuses on obesity-related insulin resistance. Adipocyte-derived exosomes participate in the activation of macrophages by promoting M1 macrophage polarization and inhibiting M2 macrophage polarization and subsequently stimulating insulin resistance. Exosomes from activated macrophages also induce insulin resistance. Furthermore, exosomes derived from tissues other than adipose tissue also play a role in obesity-related insulin resistance.

Exosomes could be potential diagnostic and therapeutic tools for insulin resistance. Liquid biopsy is a very valuable test, and it reflects the overall physical condition. Bodily fluids contain a variety of substances, so it is important to choose suitable and specific biomarkers. The exosome is a potential biomarker for insulin resistance, since it can be changed by the body’s pathophysiological state. As the exosome is a membrane structure that can protect the internal molecules from degradation, we can use exosomes to detect changes in the nucleic acid levels of patients. Although the application of exosomes as a detection method is not very common at present, with in-depth research on exosomes, people will better understand the superiority of exosomes as a tool for liquid biopsy. The application of exosomes from mesenchymal stem cells has been studied for many years. The effect of MSC-exosome treatment on insulin resistance has been confirmed in several studies. Exosomes have great advantages for the treatment of insulin resistance. Firstly, exosomes have the physiological activity of their parent cells and can replace the parent cells to play a therapeutic role. Secondly, exosomes are less immunogenic and do not cause immunologic rejection in patients. Thirdly, exosomes are vesicle structures that do not pose a tumorigenic risk to organisms. Fourthly, exosomes can treat insulin resistance by loading some drugs. Although the current research on the diagnosis and treatment of insulin resistance with exosomes is only the tip of the iceberg. In the future, using advanced technology and methods, we will skilfully use exosomes to diagnose and treat insulin resistance.

## Author Contributions

L-QY: manuscript writing and approving the final version of manuscript. L-ML: study conduct, data analysis, and manuscript writing. XL, FX, S-KS, BG, F-X-ZL, M-HZ, YW, and Q-SX: data analysis. All authors: reviewed the manuscript.

## Conflict of Interest

The authors declare that the research was conducted in the absence of any commercial or financial relationships that could be construed as a potential conflict of interest.

## Abbreviations

EVs: extracellular vesicles
ILVs: intraluminal vesicles
MVBs: multivesicular bodies
ALIX: ALG-2 (apoptosis-linked gene 2)-interacting protein X
TSG101: tumor susceptibility gene 101
Grp94: regulated protein 94
GDM: gestational diabetes mellitus
OGTT: oral glucose tolerance test
HOMA-IR: Homeostatic Model Assessment for Insulin Resistance
TNF- α: tumor necrosis factor- α
IL-6: interleukin-6
RBP4: retinol-binding protein-4
Shh: Sonic hedgehog
IRADEs: insulin resistance adipocyte-derived exosomes
IRS-1: insulin receptor substrate-1
HSL: hormone-sensitive lipase
ATM: Adipose tissue macrophage
PPAR γ: peroxisome proliferator-activated receptor γ
LPS: lipopolysaccharide
Klf4: Krüppel-like factor 4
HFD: high-fat diet
PTEN: phosphatase and tensin homolog deleted on chromosome 10
Sirt1: Sirtuin 1
FASN: fatty acid synthetase
PHLPP2: PH domain leucine-rich repeat protein phosphatase 2
GSIS: glucose-stimulated insulin secretion
eWAT: epididymal white adipose tissue
A-FABP: adipocyte fatty acid-binding protein
MSCs: mesenchymal stem cells
hucMSC-ex: exosomes from human umbilical cord mesenchymal stem cells
BM-MSCs: bone marrow mesenchymal stem cells
ADSCs: adipose-derived stem cells.

## References

[B1] Aswad H. Forterre A. Wiklander O. P. Vial G. Danty-Berger E. Jalabert A. (2014). Exosomes participate in the alteration of muscle homeostasis during lipid-induced insulin resistance in mice. *Diabetologia* 57 2155–2164. 10.1007/s00125-014-3337-2 25073444 PMC4153976

[B2] Atkins H. (2019). Stem cell transplantation to treat multiple sclerosis. *Jama* 321 153–155. 10.1001/jama.2018.20777 30644971

[B3] Ayina C. N. Noubiap J. J. Etoundi Ngoa L. S. Boudou P. Gautier J. F. Mengnjo M. K. (2016). Association of serum leptin and adiponectin with anthropomorphic indices of obesity, blood lipids and insulin resistance in a Sub-Saharan African population. *Lipids Health Dis* 15:96. 10.1186/s12944-016-0264-x 27189377 PMC4869296

[B4] Babuta M. Furi I. Bala S. Bukong T. N. Lowe P. Catalano D. (2019). Dysregulated autophagy and lysosome function are linked to exosome production by Micro-RNA 155 in alcoholic liver disease. *Hepatology* 70 2123–2141. 10.1002/hep.30766 31090940 PMC7453183

[B5] Benhalima K. Van Crombrugge P. Moyson C. Verhaeghe J. Vandeginste S. Verlaenen H. (2019). Characteristics and pregnancy outcomes across gestational diabetes mellitus subtypes based on insulin resistance. *Diabetologia* 62 2118–2128. 10.1007/s00125-019-4961-7 31338546

[B6] Bhagirath D. Yang T. L. Bucay N. Sekhon K. Majid S. Shahryari V. (2018). microRNA-1246 Is an exosomal biomarker for aggressive prostate cancer. *Cancer Res.* 78 1833–1844. 10.1158/0008-5472.Can-17-2069 29437039 PMC5890910

[B7] Carayon K. Chaoui K. Ronzier E. Lazar I. Bertrand-Michel J. Roques V. (2011). Proteolipidic composition of exosomes changes during reticulocyte maturation. *J. Biol. Chem.* 286 34426–34439. 10.1074/jbc.M111.257444 21828046 PMC3190795

[B8] Castaño C. Kalko S. Novials A. Párrizas M. (2018). Obesity-associated exosomal miRNAs modulate glucose and lipid metabolism in mice. *Proc. Natl. Acad. Sci. U.S.A.* 115 12158–12163. 10.1073/pnas.1808855115 30429322 PMC6275521

[B9] Chew J. R. J. Chuah S. J. Teo K. Y. W. Zhang S. Lai R. C. Fu J. H. (2019). Mesenchymal stem cell exosomes enhance periodontal ligament cell functions and promote periodontal regeneration. *Acta Biomater.* 89 252–264. 10.1016/j.actbio.2019.03.021 30878447

[B10] Choi Y. Kwon Y. Kim D. K. Jeon J. Jang S. C. Wang T. (2015). Gut microbe-derived extracellular vesicles induce insulin resistance, thereby impairing glucose metabolism in skeletal muscle. *Sci. Rep.* 5:15878. 10.1038/srep15878 26510393 PMC4625370

[B11] Czech M. P. (2020). Mechanisms of insulin resistance related to white, beige, and brown adipocytes. *Mol. Metab.* 34 27–42. 10.1016/j.molmet.2019.12.014 32180558 PMC6997501

[B12] Dang S. Y. Leng Y. Wang Z. X. Xiao X. Zhang X. Wen T. (2019). Exosomal transfer of obesity adipose tissue for decreased miR-141-3p mediate insulin resistance of hepatocytes. *Int. J. Biol. Sci.* 15 351–368. 10.7150/ijbs.28522 30745826 PMC6367552

[B13] Day S. E. Coletta R. L. Kim J. Y. Garcia L. A. Campbell L. E. Benjamin T. R. (2017). Potential epigenetic biomarkers of obesity-related insulin resistance in human whole-blood. *Epigenetics* 12 254–263. 10.1080/15592294.2017.1281501 28106509 PMC5398771

[B14] De Silva N. Samblas M. Martinez J. A. Milagro F. I. (2018). Effects of exosomes from LPS-activated macrophages on adipocyte gene expression, differentiation, and insulin-dependent glucose uptake. *J. Physiol. Biochem.* 74 559–568. 10.1007/s13105-018-0622-4 29560554

[B15] Deng Z. B. Poliakov A. Hardy R. W. Clements R. Liu C. Liu Y. (2009). Adipose tissue exosome-like vesicles mediate activation of macrophage-induced insulin resistance. *Diabetes* 58 2498–2505. 10.2337/db09-0216 19675137 PMC2768161

[B16] Dinh P. C. Paudel D. Brochu H. Popowski K. D. Gracieux M. C. Cores J. (2020). Inhalation of lung spheroid cell secretome and exosomes promotes lung repair in pulmonary fibrosis. *Nat. Commun.* 11:1064. 10.1038/s41467-020-14344-7 32111836 PMC7048814

[B17] Fang J. H. Zhang Z. J. Shang L. R. Luo Y. W. Lin Y. F. Yuan Y. (2018). Hepatoma cell-secreted exosomal microRNA-103 increases vascular permeability and promotes metastasis by targeting junction proteins. *Hepatology* 68 1459–1475. 10.1002/hep.29920 29637568

[B18] Friedrichsen M. Poulsen P. Richter E. A. Hansen B. F. Birk J. B. Ribel-Madsen R. (2010). Differential aetiology and impact of phosphoinositide 3-kinase (PI3K) and Akt signalling in skeletal muscle on in vivo insulin action. *Diabetologia* 53 1998–2007. 10.1007/s00125-010-1795-8 20512309

[B19] Frithioff-Bojsoe C. Lund M. A. V. Lausten-Thomsen U. Hedley P. L. Pedersen O. Christiansen M. (2020). Leptin, adiponectin, and their ratio as markers of insulin resistance and cardiometabolic risk in childhood obesity. *Pediatr. Diabetes* 21 194–202. 10.1111/pedi.12964 31845423

[B20] Fu X. Dong B. Tian Y. Lefebvre P. Meng Z. Wang X. (2015). MicroRNA-26a regulates insulin sensitivity and metabolism of glucose and lipids. *J. Clin. Invest.* 125 2497–2509. 10.1172/JCI75438 25961460 PMC4497741

[B21] Gaddam S. Periasamy R. Gangaraju R. (2019). Adult stem cell therapeutics in diabetic retinopathy. *Int. J. Mol. Sci.* 20:4876. 10.3390/ijms20194876 31575089 PMC6801872

[B22] Gemel J. Kilkus J. Dawson G. Beyer E. C. (2019). Connecting exosomes and connexins. *Cancers* 11:476. 10.3390/cancers11040476 30987321 PMC6520873

[B23] Guay C. Kruit J. K. Rome S. Menoud V. Mulder N. L. Jurdzinski A. (2019). Lymphocyte-derived exosomal MicroRNAs promote pancreatic beta cell death and may contribute to type 1 diabetes development. *Cell Metab.* 29 348.e6–361.e6. 10.1016/j.cmet.2018.09.011 30318337

[B24] Guo M. Li C. Lei Y. Xu S. Zhao D. Lu X. Y. (2017). Role of the adipose PPARgamma-adiponectin axis in susceptibility to stress and depression/anxiety-related behaviors. *Mol. Psychiatry* 22 1056–1068. 10.1038/mp.2016.225 27956741 PMC5468488

[B25] Gustafson B. Hedjazifar S. Gogg S. Hammarstedt A. Smith U. (2015). Insulin resistance and impaired adipogenesis. *Trends Endocrinol. Metab.* 26 193–200. 10.1016/j.tem.2015.01.006 25703677

[B26] Halperin F. Lopez X. Manning R. Kahn C. R. Kulkarni R. N. Goldfine A. B. (2012). Insulin augmentation of glucose-stimulated insulin secretion is impaired in insulin-resistant humans. *Diabetes* 61 301–309. 10.2337/db11-1067 22275085 PMC3266415

[B27] Hannafon B. N. Trigoso Y. D. Calloway C. L. Zhao Y. D. Lum D. H. Welm A. L. (2016). Plasma exosome microRNAs are indicative of breast cancer. *Breast. Cancer Res.* 18:90. 10.1186/s13058-016-0753-x 27608715 PMC5016889

[B28] Honkala S. M. Johansson J. Motiani K. K. Eskelinen J. J. Virtanen K. A. Loyttyniemi E. (2018). Short-term interval training alters brain glucose metabolism in subjects with insulin resistance. *J. Cereb. Blood Flow Metab.* 38 1828–1838. 10.1177/0271678X17734998 28959911 PMC6168908

[B29] Huang C. L. Wu Y. W. Hsieh A. R. Hung Y. H. Chen W. J. Yang W. S. (2013). Serum adipocyte fatty acid-binding protein levels in patients with critical illness are associated with insulin resistance and predict mortality. *Crit. Care* 17:R22. 10.1186/cc12498 23375099 PMC4056759

[B30] Hubal M. J. Nadler E. P. Ferrante S. C. Barberio M. D. Suh J. H. Wang J. (2017). Circulating adipocyte-derived exosomal MicroRNAs associated with decreased insulin resistance after gastric bypass. *Obesity* 25 102–110. 10.1002/oby.21709 27883272 PMC5182153

[B31] Inubushi S. Kawaguchi H. Mizumoto S. Kunihisa T. Baba M. Kitayama Y. (2020). Oncogenic miRNAs identified in tear exosomes from metastatic breast cancer patients. *Anticancer Res.* 40 3091–3096. 10.21873/anticanres.14290 32487603

[B32] Jia G. DeMarco V. G. Sowers J. R. (2016). Insulin resistance and hyperinsulinaemia in diabetic cardiomyopathy. *Nat. Rev. Endocrinol.* 12 144–153. 10.1038/nrendo.2015.216 26678809 PMC4753054

[B33] Jones A. Danielson K. M. Benton M. C. Ziegler O. Shah R. Stubbs R. S. (2017). miRNA signatures of insulin resistance in obesity. *Obesity* 25 1734–1744. 10.1002/oby.21950 28834285 PMC5614819

[B34] Kamalden T. A. Macgregor-Das A. M. Kannan S. M. Dunkerly-Eyring B. Khaliddin N. Xu Z. (2017). Exosomal MicroRNA-15a transfer from the pancreas augments diabetic complications by inducing oxidative stress. *Antioxid Redox Signal.* 27 913–930. 10.1089/ars.2016.6844 28173719 PMC5649125

[B35] Kapogiannis D. Boxer A. Schwartz J. B. Abner E. L. Biragyn A. Masharani U. (2015). Dysfunctionally phosphorylated type 1 insulin receptor substrate in neural-derived blood exosomes of preclinical Alzheimer’s disease. *FASEB J.* 29 589–596. 10.1096/fj.14-262048 25342129 PMC4314222

[B36] Kapustin A. N. Chatrou M. L. Drozdov I. Zheng Y. Davidson S. M. Soong D. (2015). Vascular smooth muscle cell calcification is mediated by regulated exosome secretion. *Circ. Res.* 116 1312–1323. 10.1161/CIRCRESAHA.116.305012 25711438

[B37] Katayama M. Wiklander O. P. B. Fritz T. Caidahl K. El-Andaloussi S. Zierath J. R. (2019). Circulating exosomal miR-20b-5p Is Elevated in Type 2 diabetes and could impair insulin action in human skeletal muscle. *Diabetes* 68 515–526. 10.2337/db18-0470 30552111

[B38] Kobayashi Y. Eguchi A. Tempaku M. Honda T. Togashi K. Iwasa M. (2018). Circulating extracellular vesicles are associated with lipid and insulin metabolism. *Am. J. Physiol. Endocrinol. Metab.* 315 E574–E582. 10.1152/ajpendo.00160.2018 29944389

[B39] Kranendonk M. E. Visseren F. L. van Herwaarden J. A. Nolte-’t Hoen E. N. de Jager W. Wauben M. H. (2014). Effect of extracellular vesicles of human adipose tissue on insulin signaling in liver and muscle cells. *Obesity* 22 2216–2223. 10.1002/oby.20847 25045057

[B40] Lasser C. Alikhani V. S. Ekstrom K. Eldh M. Paredes P. T. Bossios A. (2011). Human saliva, plasma and breast milk exosomes contain RNA: uptake by macrophages. *J. Transl. Med.* 9:9. 10.1186/1479-5876-9-9 21235781 PMC3033821

[B41] Li F. Li H. Jin X. Zhang Y. Kang X. Zhang Z. (2019). Adipose-specific knockdown of Sirt1 results in obesity and insulin resistance by promoting exosomes release. *Cell Cycle* 18 2067–2082. 10.1080/15384101.2019.1638694 31296102 PMC6681786

[B42] Li X. Zhu Q. Wang W. Qi J. He Y. Wang Y. (2019). Elevated chemerin induces insulin resistance in human granulosa-lutein cells from polycystic ovary syndrome patients. *Faseb J.* 33 11303–11313. 10.1096/fj.201802829R 31311314

[B43] Li G. Esangbedo I. C. Xu L. Fu J. Li L. Feng D. (2018). Childhood retinol-binding protein 4 (RBP4) levels predicting the 10-year risk of insulin resistance and metabolic syndrome: the BCAMS study. *Cardiovasc. Diabetol.* 17:69. 10.1186/s12933-018-0707-y 29759068 PMC5950249

[B44] Li W. Li C. Zhou T. Liu X. Liu X. Li X. (2017). Role of exosomal proteins in cancer diagnosis. *Mol. Cancer* 16:145. 10.1186/s12943-017-0706-8 28851367 PMC5576100

[B45] Liang X. Ding Y. Zhang Y. Tse H. F. Lian Q. (2014). Paracrine mechanisms of mesenchymal stem cell-based therapy: current status and perspectives. *Cell Transplant* 23 1045–1059. 10.3727/096368913x667709 23676629

[B46] Liu T. Sun Y. C. Cheng P. Shao H. G. (2019). Adipose tissue macrophage-derived exosomal miR-29a regulates obesity-associated insulin resistance. *Biochem. Biophys. Res. Commun.* 515 352–358. 10.1016/j.bbrc.2019.05.113 31153636

[B47] Liu X. Miao J. Wang C. Zhou S. Chen S. Ren Q. (2020). Tubule-derived exosomes play a central role in fibroblast activation and kidney fibrosis. *Kidney Int.* 97 1181–1195. 10.1016/j.kint.2019.11.026 32139089

[B48] Lou G. Chen Z. Zheng M. Liu Y. (2017). Mesenchymal stem cell-derived exosomes as a new therapeutic strategy for liver diseases. *Exp. Mo. Med.* 49 e346–e346. 10.1038/emm.2017.63 28620221 PMC5519012

[B49] Lu M. Yuan S. Li S. Li L. Liu M. Wan S. (2019). The exosome-derived biomarker in atherosclerosis and its clinical application. *J. Cardiovasc. Transl. Res.* 12 68–74. 10.1007/s12265-018-9796-y 29802541

[B50] Mauer J. Chaurasia B. Goldau J. Vogt M. C. Ruud J. Nguyen K. D. (2014). Signaling by IL-6 promotes alternative activation of macrophages to limit endotoxemia and obesity-associated resistance to insulin. *Nat. Immunol.* 15 423–430. 10.1038/ni.2865 24681566 PMC4161471

[B51] Mazur-Bialy A. I. (2019). Superiority of the non-glycosylated form over the glycosylated form of irisin in the attenuation of adipocytic meta-inflammation: a potential factor in the fight against insulin resistance. *Biomolecules* 9:394. 10.3390/biom9090394 31438646 PMC6770638

[B52] McBride J. D. Rodriguez-Menocal L. Guzman W. Candanedo A. Garcia-Contreras M. Badiavas E. V. (2017). Bone marrow mesenchymal stem cell-derived CD63(+) exosomes transport wnt3a exteriorly and enhance dermal fibroblast proliferation. Migration, and Angiogenesis In Vitro. *Stem Cells Dev.* 26 1384–1398. 10.1089/scd.2017.0087 28679315

[B53] McKiernan J. Donovan M. J. Margolis E. Partin A. Carter B. Brown G. (2018). A prospective adaptive utility trial to validate performance of a novel urine exosome gene expression assay to predict high-grade prostate cancer in patients with prostate-specific Antigen 2–10 ng/ml at initial biopsy. *Eur. Urol.* 74 731–738. 10.1016/j.eururo.2018.08.019 30237023

[B54] McQuaid S. E. Hodson L. Neville M. J. Dennis A. L. Cheeseman J. Humphreys S. M. (2011). Downregulation of adipose tissue fatty acid trafficking in obesity: a driver for ectopic fat deposition? *Diabetes* 60 47–55. 10.2337/db10-0867 20943748 PMC3012196

[B55] Mleczko J. Ortega F. J. Falcon-Perez J. M. Wabitsch M. Fernandez-Real J. M. Mora S. (2018). Extracellular vesicles from hypoxic adipocytes and obese subjects reduce insulin-stimulated glucose uptake. *Mol. Nutr. Food Res.* 62:1700917. 10.1002/mnfr.201700917 29292863 PMC5887919

[B56] Mullins R. J. Mustapic M. Goetzl E. J. Kapogiannis D. (2017). Exosomal biomarkers of brain insulin resistance associated with regional atrophy in Alzheimer’s disease. *Hum. Brain Mapp.* 38 1933–1940. 10.1002/hbm.23494 28105773 PMC5342917

[B57] Mundi M. S. Koutsari C. Jensen M. D. (2014). Effects of increased free fatty acid availability on adipose tissue fatty acid storage in men. *J. Clin. Endocrinol. Metab.* 99 E2635–E2642. 10.1210/jc.2014-2690 25192251 PMC4255130

[B58] Muraro P. A. Martin R. Mancardi G. L. Nicholas R. Sormani M. P. Saccardi R. (2017). Autologous haematopoietic stem cell transplantation for treatment of multiple sclerosis. *Nat. Rev. Neurol.* 13 391–405. 10.1038/nrneurol.2017.81 28621766

[B59] Nie Y. Sato Y. Garner R. T. Kargl C. Wang C. Kuang S. (2019). Skeletal muscle-derived exosomes regulate endothelial cell functions via reactive oxygen species-activated nuclear factor-kappaB signalling. *Exp. Physiol.* 104 1262–1273. 10.1113/EP087396 31115069

[B60] Olefsky J. M. Glass C. K. (2010). Macrophages, inflammation, and insulin resistance. *Annu. Rev. Physiol.* 72 219–246. 10.1146/annurev-physiol-021909-135846 20148674

[B61] Pan Y. Hui X. Hoo R. L. C. Ye D. Chan C. Y. C. Feng T. (2019). Adipocyte-secreted exosomal microRNA-34a inhibits M2 macrophage polarization to promote obesity-induced adipose inflammation. *J. Clin. Investig.* 129 834–849. 10.1172/jci123069 30667374 PMC6355214

[B62] Seo W. Eun H. S. Kim S. Y. Yi H. S. Lee Y. S. Park S. H. (2016). Exosome-mediated activation of toll-like receptor 3 in stellate cells stimulates interleukin-17 production by gammadelta T cells in liver fibrosis. *Hepatology* 64 616–631. 10.1002/hep.28644 27178735

[B63] Sharma R. Kumari M. Prakash P. Gupta S. Tiwari S. (2020). Phosphoenolpyruvate carboxykinase in urine exosomes reflect impairment in renal gluconeogenesis in early insulin resistance and diabetes. *Am. J. Physiol. Renal. Physiol.* 318 F720–F731. 10.1152/ajprenal.00507.2019 32036699

[B64] Shem-Tov N. Peczynski C. Labopin M. Itälä-Remes M. Blaise D. Labussière-Wallet H. (2020). Haploidentical vs. unrelated allogeneic stem cell transplantation for acute lymphoblastic leukemia in first complete remission: on behalf of the ALWP of the EBMT. *Leukemia* 34 283–292. 10.1038/s41375-019-0544-3 31427719

[B65] Shim Y. S. Kang M. J. Oh Y. J. Baek J. W. Yang S. Hwang I. T. (2017). Fetuin-A as an alternative marker for insulin resistance and cardiovascular risk in prepubertal children. *J. Atherosc. Thromb.* 24 1031–1038. 10.5551/jat.38323 28154244 PMC5656765

[B66] Sohn W. Kim J. Kang S. H. Yang S. R. Cho J. Y. Cho H. C. (2015). Serum exosomal microRNAs as novel biomarkers for hepatocellular carcinoma. *Exp. Mol. Med.* 47:e184. 10.1038/emm.2015.68 26380927 PMC4650928

[B67] Song M. Han L. Chen F. F. Wang D. Wang F. Zhang L. (2018). Adipocyte-derived exosomes carrying sonic hedgehog mediate M1 macrophage polarization-induced insulin resistance via Ptch and PI3K Pathways. *Cell Physiol. Biochem.* 48 1416–1432. 10.1159/000492252 30064125

[B68] Steculorum S. M. Ruud J. Karakasilioti I. Backes H. Engstrom Ruud L. Timper K. (2016). AgRP neurons control systemic insulin sensitivity via myostatin expression in brown adipose tissue. *Cell* 165 125–138. 10.1016/j.cell.2016.02.044 27015310 PMC5157157

[B69] Stenvers D. J. Scheer F. A. J. L. Schrauwen P. la Fleur S. E. Kalsbeek A. (2018). Circadian clocks and insulin resistance. *Nat. Rev. Endocrinol.* 15 75–89. 10.1038/s41574-018-0122-1 30531917

[B70] Su T. Xiao Y. Xiao Y. Guo Q. Li C. Huang Y. (2019). Bone marrow mesenchymal stem cells-derived exosomal MiR-29b-3p regulates aging-associated insulin resistance. *ACS Nano* 13 2450–2462. 10.1021/acsnano.8b09375 30715852

[B71] Sun Y. Shi H. Yin S. Ji C. Zhang X. Zhang B. (2018). Human mesenchymal stem cell derived exosomes alleviate Type 2 diabetes mellitus by reversing peripheral insulin resistance and relieving beta-cell destruction. *ACS Nano* 12 7613–7628. 10.1021/acsnano.7b07643 30052036

[B72] Templeman N. M. Skovsø S. Page M. M. Lim G. E. Johnson J. D. (2017). A causal role for hyperinsulinemia in obesity. *J. Endocrinol.* 232 R173–R183. 10.1530/joe-16-0449 28052999

[B73] Titchenell P. M. Lazar M. A. Birnbaum M. J. (2017). Unraveling the regulation of hepatic metabolism by insulin. *Trends Endocrinol.Metab.* 28 497–505. 10.1016/j.tem.2017.03.003 28416361 PMC5477655

[B74] Tokarz V. L. MacDonald P. E. Klip A. (2018). The cell biology of systemic insulin function. *J. Cell Biol.* 217 2273–2289. 10.1083/jcb.201802095 29622564 PMC6028526

[B75] Vlavcheski F. Tsiani E. (2018). Attenuation of free fatty acid-induced muscle insulin resistance by rosemary extract. *Nutrients* 10:1623. 10.3390/nu10111623 30400151 PMC6267446

[B76] Wakabayashi T. Yamaguchi K. Matsui K. Sano T. Kubota T. Hashimoto T. (2019). Differential effects of diet- and genetically-induced brain insulin resistance on amyloid pathology in a mouse model of Alzheimer’s disease. *Mol. Neurodegen.* 14:15. 10.1186/s13024-019-0315-7 30975165 PMC6460655

[B77] Wang L. Zhang B. Zheng W. Kang M. Chen Q. Qin W. (2017). Exosomes derived from pancreatic cancer cells induce insulin resistance in C2C12 myotube cells through the PI3K/Akt/FoxO1 pathway. *Sci. Rep.* 7:5384. 10.1038/s41598-017-05541-4 28710412 PMC5511275

[B78] Willis G. R. Fernandez-Gonzalez A. Anastas J. Vitali S. H. Liu X. Ericsson M. (2018). Mesenchymal stromal cell exosomes ameliorate experimental bronchopulmonary dysplasia and restore lung function through macrophage immunomodulation. *Am. J. Respir. Crit. Care Med.* 197 104–116. 10.1164/rccm.201705-0925OC 28853608 PMC5765387

[B79] Wortzel I. Dror S. Kenific C. M. Lyden D. (2019). Exosome-mediated metastasis: communication from a distance. *Dev. Cell* 49 347–360. 10.1016/j.devcel.2019.04.011 31063754

[B80] Wu C. X. Liu Z. F. (2018). Proteomic profiling of sweat exosome suggests its involvement in skin immunity. *J. Invest. Dermatol.* 138 89–97. 10.1016/j.jid.2017.05.040 28899687

[B81] Wu J. Dong T. Chen T. Sun J. Luo J. He J. (2020). Hepatic exosome-derived miR-130a-3p attenuates glucose intolerance via suppressing PHLPP2 gene in adipocyte. *Metabolism* 103:154006. 10.1016/j.metabol.2019.154006 31715176

[B82] Xia C. Zeng Z. Fang B. Tao M. Gu C. Zheng L. (2019). Mesenchymal stem cell-derived exosomes ameliorate intervertebral disc degeneration via anti-oxidant and anti-inflammatory effects. *Free Radic. Biol. Med.* 143 1–15. 10.1016/j.freeradbiomed.2019.07.026 31351174

[B83] Xie C. Ji N. Tang Z. Li J. Chen Q. (2019). The role of extracellular vesicles from different origin in the microenvironment of head and neck cancers. *Mol. Cancer* 18:83. 10.1186/s12943-019-0985-3 30954079 PMC6451295

[B84] Xu F. Zhong J. Y. Lin X. Shan S. K. Guo B. Zheng M. H. (2020). Melatonin alleviates vascular calcification and ageing through exosomal miR-204/miR-211 cluster in a paracrine manner. *J. Pineal Res.* 68:e12631. 10.1111/jpi.12631 31943334 PMC7154654

[B85] Xu H. Du X. Xu J. Zhang Y. Tian Y. Liu G. (2020). Pancreatic beta cell microRNA-26a alleviates type 2 diabetes by improving peripheral insulin sensitivity and preserving beta cell function. *PLoS Biol.* 18:e3000603. 10.1371/journal.pbio.3000603 32092075 PMC7058362

[B86] Xu X. Krumm C. So J. S. Bare C. J. Holman C. Gromada J. (2018). Preemptive activation of the integrated stress response protects mice from diet-induced obesity and insulin resistance by fibroblast growth factor 21 induction. *Hepatology* 68 2167–2181. 10.1002/hep.30060 29698569 PMC6203669

[B87] Xue M. Chen W. Xiang A. Wang R. Chen H. Pan J. (2017). Hypoxic exosomes facilitate bladder tumor growth and development through transferring long non-coding RNA-UCA1. *Mol. Cancer* 16:143. 10.1186/s12943-017-0714-8 28841829 PMC5574139

[B88] Ying W. Riopel M. Bandyopadhyay G. Dong Y. Birmingham A. Seo J. B. (2017). Adipose tissue macrophage-derived exosomal miRNAs can modulate in vivo and in vitro insulin sensitivity. *Cell* 171 372.e12–384.e12. 10.1016/j.cell.2017.08.035 28942920

[B89] Yu Y. Du H. Wei S. Feng L. Li J. Yao F. (2018). Adipocyte-derived exosomal MiR-27a induces insulin resistance in skeletal muscle through repression of PPARγ. *Theranostics* 8 2171–2188. 10.7150/thno.22565 29721071 PMC5928879

[B90] Zeng Z. Li Y. Pan Y. Lan X. Song F. Sun J. (2018). Cancer-derived exosomal miR-25-3p promotes pre-metastatic niche formation by inducing vascular permeability and angiogenesis. *Nat. Commun.* 9:5395. 10.1038/s41467-018-07810-w 30568162 PMC6300604

[B91] Zhang Y. Kim M. S. Jia B. Yan J. Zuniga-Hertz J. P. Han C. (2017). Hypothalamic stem cells control ageing speed partly through exosomal miRNAs. *Nature* 548 52–57. 10.1038/nature23282 28746310 PMC5999038

[B92] Zhao H. Shang Q. Pan Z. Bai Y. Li Z. Zhang H. (2018). Exosomes from adipose-derived stem cells attenuate adipose inflammation and obesity through polarizing M2 macrophages and beiging in white adipose tissue. *Diabetes* 67 235–247. 10.2337/db17-0356 29133512

[B93] Zhao L. Chen S. Shi X. Cao H. Li L. (2018). A pooled analysis of mesenchymal stem cell-based therapy for liver disease. *Stem Cell Res. Ther.* 9:72. 10.1186/s13287-018-0816-2 29562935 PMC5863358

[B94] Zhao Y. Xu J. (2018). Synovial fluid-derived exosomal lncRNA PCGEM1 as biomarker for the different stages of osteoarthritis. *Int. Orthop.* 42 2865–2872. 10.1007/s00264-018-4093-6 30128669

